# Enhancing Skin Rejuvenation: Using of Engineered Exosome Content Treated With Oleuropein and Fe_3_O_4_ @CQD/Oleuropein

**DOI:** 10.1111/jocd.70351

**Published:** 2025-07-14

**Authors:** Naeimeh Safavizadeh, Zahra Noormohammadi, Mohammad Zaefizadeh, Kazem Nejati‐Koshki

**Affiliations:** ^1^ Department of Biology, Science and Research Branch Islamic Azad University Tehran Iran; ^2^ Department of Biology, Ardabil Branch Islamic Azad University Ardabil Iran; ^3^ Pharmaceutical Sciences Research Center Ardabil University of Medical Sciences Ardabil Iran

**Keywords:** Fe_3_O_4_@CQD/oleuropein, microRNAs, modified exosomal content, oleuropein, skin rejuvenation

## Abstract

**Background:**

Skin aging, which is affected by intrinsic and extrinsic factors, leads to reduced elastin, collagen, and hydration levels.

**Aims:**

This study aimed to utilize modified exosomal content with treated Oleuropein and Fe_3_O_4_@C/Oleuropein to modulate gene and microRNA expression on the HFFF2 cells in vitro in order to reduce skin aging.

**Material & Methods:**

Fe_3_O_4_@C/Oleuropein was synthesized using the hydrothermal method and confirmed by XRD, FTIR, and SEM. The MTT assay was conducted to test toxicity Following this, hUC‐MSCs and HFFF2 cells were treated with Fe3O4@C/Oleuropein and Oleuropein. Exosomes derived from the treatments were extracted by ultracentrifugation and evaluated by DLS and western blotting. HFFF2 cells were treated with exosomes derived from the treatments. The expression of the studied genes and related microRNAs was measured using qRT‐PCR. Also, the effect of exosomes derived from the treatments on HFFF2 cells was evaluated using flow cytometry.

**Results:**

The results showed that the expression of *IGF1*, *IGF1R*, *COL1A1*, *ELN*, and *EGF* genes was significantly increased with Oleuropein (500 μg/mL) and Fe_3_O_4_@C/Oleuropein (250 μg/mL) treatments, especially when treated with exosomes derived from treatments. Moreover, the expression of *hsa‐miR‐29b‐3p*, *hsa‐let‐7d‐5p*, and *hsa‐let‐7e‐5p* microRNAs was significantly downregulated, and *hsa‐miR‐34a‐5p* was upregulated in the HFFF2 cells, which was consistent with the exosome cargo derived from treated cells.

**Conclusions:**

Exosomes can increase gene expression and reduce microRNAs associated with skin antiaging. Using modified exosomal content treated with Oleuropein and Fe_3_O_4_@CQD/Oleuropein is generally effective for preventing skin aging and also presents innovative methods for skin care.

## 1 Introduction

Currently, there is growing attention to skin rejuvenation because of the increase in people's life expectancy [1]. Skin aging is a complicated process that includes intrinsic (chronological) aging and extrinsic aging (e.g., sun, gravity, sleeping positions, and smoking). In both types of aging, there is a decrease in elastin, collagen, and loss in hydration leading to dryness and atrophy, with fine wrinkling in the skin [2, 3]. Fibroblasts, the predominant population responsible for extracellular matrix (ECM) organization, are the primary cause of skin senescence [4, 5].

So far, researchers have extensively studied skin rejuvenation and proposed strategies for reducing skin aging, one of which is nanotechnology. Recent research shows that Fe_3_O_4_@CQDs significantly reduce ROS and inhibit pro‐inflammatory cytokines like *TNF‐α* and *IL‐1β*, addressing oxidative damage and aging pathways [6, 7]. Studies indicate that nanotechnology enhances cosmetic effectiveness by increasing capture efficiency, controlling drug release, improving physical stability, increasing moisturization, and enhancing ultraviolet (UV) protection [8].

As more people become interested in natural cosmetics, Oleuropein, a phenolic substance found in olive leaves ( *Olea europaea* ), has been shown to have multiple skin advantages in clinical studies [9, 10, 11]. This secoiridoid has a special ability to change how different mechanisms of skin aging interact, such as oxidative stress, inflammasome activation, and the degeneration of the ECM [12, 13]. Studies show that it is able to eliminate reactive oxygen species (ROS), lower the levels of pro‐inflammatory cytokines including *IL‐1β* and *IL‐6*, and boost collagen production through pathways that are mediated by *VEGF* [14, 15]. Oleuropein increases collagen synthesis and inhibits matrix metalloproteinases (*MMPs*), which degrade skin collagen and elastin [10, 11].

Also, studies have shown that vitamin C is one of the compounds that affects skin rejuvenation. Vitamin C neutralizes free radicals and increases collagen, but Oleuropein does better in the role of removing ROS [16] while also inhibiting *IL‐1β* and *caspase‐1*‐mediated inflammasome activation, which is associated with both oxidative stress and inflammation [16]. Oleuropein is stable in formulations, unlike vitamin C, which degrades quickly [17]. Based on research, Retinol enhances skin texture but induces inflammation and necessitates the avoidance of UV exposure, whereas Fe_3_O_4_@CQDs decrease inflammation without impairing barrier function, and their magnetic targeting minimizes systemic exposure [18, 19]. In contrast to hyaluronic acid or niacinamide, which disperse widely, Fe_3_O_4_@CQDs improve spatial accuracy by concentrating active ingredients in photoaged or inflammatory regions [8, 20, 21].

Another strategy for decreasing skin aging is exosomes, which have recently attracted significant attention as a therapy for skin rejuvenation [22]. Exosomes, a category of skincare agents, are increasing in popularity in the cosmetics industry because of their therapeutic and rejuvenating characteristics. They contain proteins, lipids, nucleic acids like microRNAs, and other compounds, which promote healing, hydration, and skin health. Exosomes can be modified by manipulating the culture medium or cell content and can repair skin damage such as sunburn and acne scars. They also contain bioactive compounds that protect the skin from environmental influences and minimize dark spots [23, 24, 25, 26]. On the other hand, microRNAs are one of the most major molecules present in exosomes [27]. MicroRNAs have been shown to have various activities in skin aging after differentially expressed microRNAs were identified based on the results of microRNA sequence analysis [28].

Recent clinical trials and research have shown that both unmodified and modified (engineered) exosomes, particularly those sourced from mesenchymal stem cells (MSCs) and fibroblasts, are efficacious in skin rejuvenation, wound healing, and enhancement of barrier function [29, 30, 31]. Also, exosome‐based creams represent an innovative method in cosmetic dermatology, proving efficacy in skin rejuvenation, hydration, pigmentation correction, and wound healing [31, 32].

Research findings suggest that multiple signaling pathways play a role in the process of skin aging. Insulin‐like growth factor 1 (*IGF1*) is a key protein that stimulates keratinocyte growth [4]. Many downstream signaling pathways, including PI3K/AKT and MAPK, are phosphorylated as a result of *IGF1* activation of the insulin‐like growth factor 1 receptor (*IGF1R*) [33]. *IGF1* activates the PI3PK/AKT pathway, prevents dermal fibroblasts from UV‐B‐induced programmed cell death, and promotes cell survival via the MAPK pathway [34]. Also, epidermal growth factor (*EGF*) is a mitogenic polypeptide. It can activate and multiply myofibroblasts through the ERK/MAPK and PI3K/AKT signaling pathways [35, 36]. *EGF* has a bright future in cosmetic antiaging applications because it plays an important role in upregulating fibroblast proliferation and collagen synthesis in the skin [37].

The present study aimed to utilize modified exosomal content with treated Oleuropein and Fe3O4@C/Oleuropein to modulate the expression of genes (*IGF1*, *IGF1R*, *EGF*, *COL1A1*, and *ELN*) and microRNAs (*hsa‐miR‐29b‐3p*, *hsa‐let‐7d‐5p*, *hsa‐let‐7e‐5p*, and *hsa‐miR‐34a‐5p*) related to IGF1, EGFR, MAPK, and PI3K pathways on the HFFF2 cells (in vitro) in order to reduce the aging process in the skin.

## 2 Materials and Methods

### 2.1 Fe_3_O_4_ @CQD/Oleuropein Synthesis

Fe_3_O_4_ (Cas No 1309‐37‐1; US Research Nanomaterials Inc., USA) nanoparticles (1 g) and d‐glucose (0.5 g: a compound of carbon origin) were suspended in 60 mL of distilled water, and the suspension was sonicated for 30 min. Subsequently, using the hydrothermal method, the suspension was incubated at 180°C for 5 h to synthesize Fe_3_O_4_@CQD in the Hydrothermal Autoclave. Thereafter, the synthesized Fe_3_O_4_@CQD was separated by centrifugation at 2000 *g* for min. After washing with water and ethanol, the samples were dried using a freeze dryer. In the following, 1 g of dried Fe_3_O_4_@CQD and 0.1 g of Oleuropein (32619‐42‐4; Sigma‐Aldrich, Germany) were suspended in 50 mL of distilled water and stirred by a shaker for 24 h to make Fe_3_O_4_@CQD/Oleuropein. Finally, the Fe@C/Ole NPs were gathered, washed, and dried using a freeze dryer.

#### 2.1.1 Characteristics of Fe_3_O_4_ @CQD/Oleuropein Synthesis

The synthesized Fe@C/Ole and functional groups of the particles were characterized using Fourier Transform Infrared (FT‐IR) spectroscopy. The assay was carried out using an FTIR (SHIMADZU, Japan) device in a wave range of 500–4000 cm^−1^. X‐ray Diffraction (XRD) analysis was used to assess the physical phase and crystal structure of Fe@C/Ole (CuKα, radiation, λ = 0.154056) at a scanning speed of 2° per minute (2θ range from 10° to 80°). Additionally, the size range, morphology, and aggregation level of the synthesized Fe@C/Ole were examined by scanning electron microscopy (SEM) (TESCAN MiRa3).

### 2.2 Cell Culture

Human umbilical cord mesenchymal cells (hUC‐MSCs) were cultured according to Maleki et al. method [38]. Also, the fibroblast cell line (HFFF2) was purchased from the National Cell Bank of Iran (NCBI; Pasteur Institute, Tehran, Iran). Cell culture was performed using LG‐DMEM (Biosera, France), 10% FBS (Gibco, USA), Pen/Strep 100 μg/mL (Biosera, France), and then hUC‐MSCs and HFFF2 cells were incubated in 96% humidity containing 5% CO_2_ at 37°C. hUC‐MSCs and HFFF2 cells were subcultured respectively after 80% and 90% confluency using 0.25% trypsin/EDTA (Gibco, USA).

### 2.3 Cell Viability Assay (MTT Assay)

hUC‐MSCs and HFFF2 cells suspensions were seeded in a 96‐well culture plate, and after incubation for 24 h, hUC‐MSCs and HFFF2 cells were treated with a gradient concentration of Fe@C/Ole (0–1000 μg/mL) and Ole (0–2000 μg/mL). The plates were incubated for 24 and 48 h. Next, the medium was removed, and MTT (2‐(4,5‐dimethythiazol‐2‐yl)‐2,5‐diphenyltetrazolium bromide) solution (Sigma Aldrich, USA) was added. After incubation for 4 h, the contents of the wells were removed and dimethyl sulfoxide (DMSO) was added to the wells (the assay was repeated three times). The IC_50_ and IC_25_ were calculated by measuring the absorbance of the formazan crystals at a wavelength of 570 nm (Awareness Microwell Plate Reader Chromate 4300, USA).

### 2.4 Treatment of Cells With Fe@C/Ole and Ole

The hUC‐MSCs and HFFF2 cells were seeded for 24 h before treatment in culture flasks. The next day, the hUC‐MSCs and HFFF2 cells were treated with Fe@C/Ole (IC_25_ ≃ 250 μg/mL) and Ole (IC_25_ ≃ 500 μg/mL) for 24 h; IC_25_ was utilized to reduce damage and maintain the safety of the cells.

### 2.5 Exosomes Isolation and Characterization

#### 2.5.1 Exosomes Isolation

The cell culture supernatants were collected and transferred into centrifuge microtubes after 24 h of treatment with Fe@C/Ole and Ole. We extracted exosomes from exosomes isolated from cell cultures by ultracentrifugation using optimized protocols (Thermo Fisher Scientific, USA). In summary, the supernatants were centrifuged in the following order: 300 *g*, 2000 *g*, 10 min each, at 4°C, and 10 000 *g* for 30 min at 4°C. The supernatants obtained were ultracentrifuged at 100 000 *g* for 90 min at 4°C. Next, the pellets were resuspended in PBS and ultracentrifuged at 100 000 *g* for 90 min at 4°C. Finally, the pellets were resuspended in 500 μL of PBS and stored at −80°C.

#### 2.5.2 Exosome Characterization Assays

##### 2.5.2.1 Dynamic Light Scattering (DLS)

The sizes of the isolated hUC‐MSC‐derived exosomes and HFFF2‐derived exosomes resuspended in PBS were measured using Dynamic Light Scattering (DLS) (Horiba‐SZ‐100Z, Japan), employing the following parameters: viscosity, 0.894 mPa·s; refractive index, 1.330.

##### 2.5.2.2 Western Blot Analysis

Isolated hUC‐MSC‐derived exosomes (500 μL) and HFFF2‐derived exosomes resuspended in PBS were lysed in a lysis buffer. After centrifuging at 12 000 *g* for 12 min at 4°C, the supernatant containing protein was extracted and stored in a freezer at −20°C. Protein concentration was measured using the Bradford assay. Quantified proteins were separated on a 10% polyacrylamide gel and electrotransferred to polyvinylidene fluoride (PVDF, Thermo Scientific, USA) membranes. The membranes were blocked with 5% BSA (Roche, Germany) for 2 h and then incubated with primary antibodies against CD63 (Santa Cruz, USA, #sc‐5275), CD9 (Santa Cruz, USA, #sc‐18 869), and β‐actin (Santa Cruz, USA, #sc‐47 778) for 18 h. After washing, the membranes were incubated for 1:15 h 15 min with a secondary anti‐rabbit antibody (Santa Cruz, USA, #sc‐2357). Enhanced chemiluminescence (ECL) was used to detect the bands, and ImageJ software was used for analysis.

### 2.6 Treatment of Cells With Isolated Exosomes

The HFFF2 cells were seeded for 24 h before treatment in culture flasks. The next day, the HFFF2 cells were treated with hUC‐MSC‐derived exosomes and HFFF2‐derived exosomes (at Bradford assay concentration) for 24 h.

### 2.7 Prediction of microRNAs Related to Studied Genes

In silico studies were employed to confirm the selected microRNAs associated with studied genes utilizing various algorithms, including TargetScan, miRDB, miRTarBase, miRNet, miRbase, BiBiServ2‐RNAhybrid, and the Ensembl genome browser 112 databases.

### 2.8 Gene and microRNA Expression Analysis by qRT‐PCR

Total RNA was extracted using EX6101‐RNX Plus Solution (SinaClone, Iran) at two levels: level 1. hUC‐MSCs and HFFF2 cells treated with Fe@C/Ole, Ole, and control groups (at IC_25_ concentration) for 24 h, and level 2. HFFF2 cells treated with hUC‐MSC‐derived exosomes, HFFF2‐derived exosomes, and control groups (at Bradford assay concentration) for 24 h. Additionally, microRNAs were isolated using the RNeasy Mini Kit (Qiagen, Germany) following the manufacturer's protocol at two levels: level 1. hUC‐MSC‐derived exosomes and HFFF2‐derived exosomes treated with Fe@C/Ole, Ole, and control groups (at IC_25_ concentration) for 24 h, and level 2. HFFF2 cells treated with hUC‐MSC‐derived exosomes, HFFF2‐derived exosomes, and control groups (at Bradford assay concentration) for 24 h. The genomic DNA‐free RNA was quantified by the absorption 260/280 nm Nanodrop (Thermo Fisher Scientific, USA). Then, cDNA was synthesized using the SinaClon First Strand cDNA Synthesis Kit (SinaClone, Iran) from total extracted RNAs according to the manufacturer's protocol; also, cDNA of extracted microRNAs was synthesized utilizing Stem Loop specific primers according to the instructions of the SinaClon First Strand cDNA Synthesis Kit (SinaClone, Iran).

The relative expression of *IGF1*, *IGF1R*, *COL1A1*, *ELN*, and *EGF* was analyzed by qRT‐PCR using the gene‐specific primers shown in Table S1. The relative expression of these genes was examined at two levels: level 1. hUC‐MSCs and HFFF2 cells treated with Fe@C/Ole, Ole, and control groups, and level 2. HFFF2 cells treated with hUC‐MSC‐derived exosomes, HFFF2‐derived exosomes, and control groups. On the other hand, the relative expression of *hsa‐miR‐29b‐3p*, *hsa‐miR‐34a‐5p*, *hsa‐let‐7d‐5p*, and *hsa‐let‐7e‐5p* microRNAs was examined using the microRNA‐specific primers presented in Table S2. The relative expression of these microRNAs was analyzed at two levels: level 1. hUC‐MSC‐derived exosomes, HFFF2‐derived exosomes treated with Fe@C/Ole, Ole, and control groups, and level 2. HFFF2 cells treated with hUC‐MSC‐derived exosomes, HFFF2‐derived exosomes, and control groups. Gene amplification was performed using the Sina SYBR Blue HS‐qPCR kit (SinaClone, Iran), in accordance with the manufacturer's protocol. Relative analysis at the mRNA and microRNA levels was performed with two replicates using the *Pfaffl* method with $e^{-\Delta \Delta C_{t}}$ compared to the *GAPDH* and *U6* housekeeping genes, respectively.

### 2.9 Flow Cytometry (Exosome Toxicity Investigation)

Cells were harvested and stained with DAPI 10X (4′,6‐diamidino‐2‐phenylindole). All tests were performed in triplicate. The cell cycle and apoptosis progression were measured using an automated multicolor Flow Cytometry system. Flow Cytometry results were evaluated using FlowJo software (version 10). Flow Cytometry was used to measure the apoptosis rate in the HFFF2 cells treated with hUC‐MSC‐derived exosomes and HFFF2‐derived exosomes treated with Fe@C/Ole, Ole, and control groups. The HFFF2 cells were treated with two different concentrations of exosomes: 5.71 μg/mL (Bradford assay concentration) for HFFF2‐derived exosomes treated with Fe@C/Ole, Ole, and control groups, and 6.088 μg/mL (Bradford assay concentration) for hUC‐MSC‐derived exosomes treated with Fe@C/Ole, Ole, and control groups.

### 2.10 Statistical Analysis

Analysis of variance (ANOVA) was applied using relative gene expression data, and post hoc mean comparison was performed using Tukey's method test at *p ≤ 0.01* or *p ≤ 0.05*. Statistical analysis of the data and diagrams were performed using Prismv10.3.1.509 software (GraphPad Software Inc., La Jolla, CA, USA).

## 3 Results

### 3.1 Physicochemical Properties of Fe_3_O_4_ @CQD/Oleuropein

**FIGURE 1:**
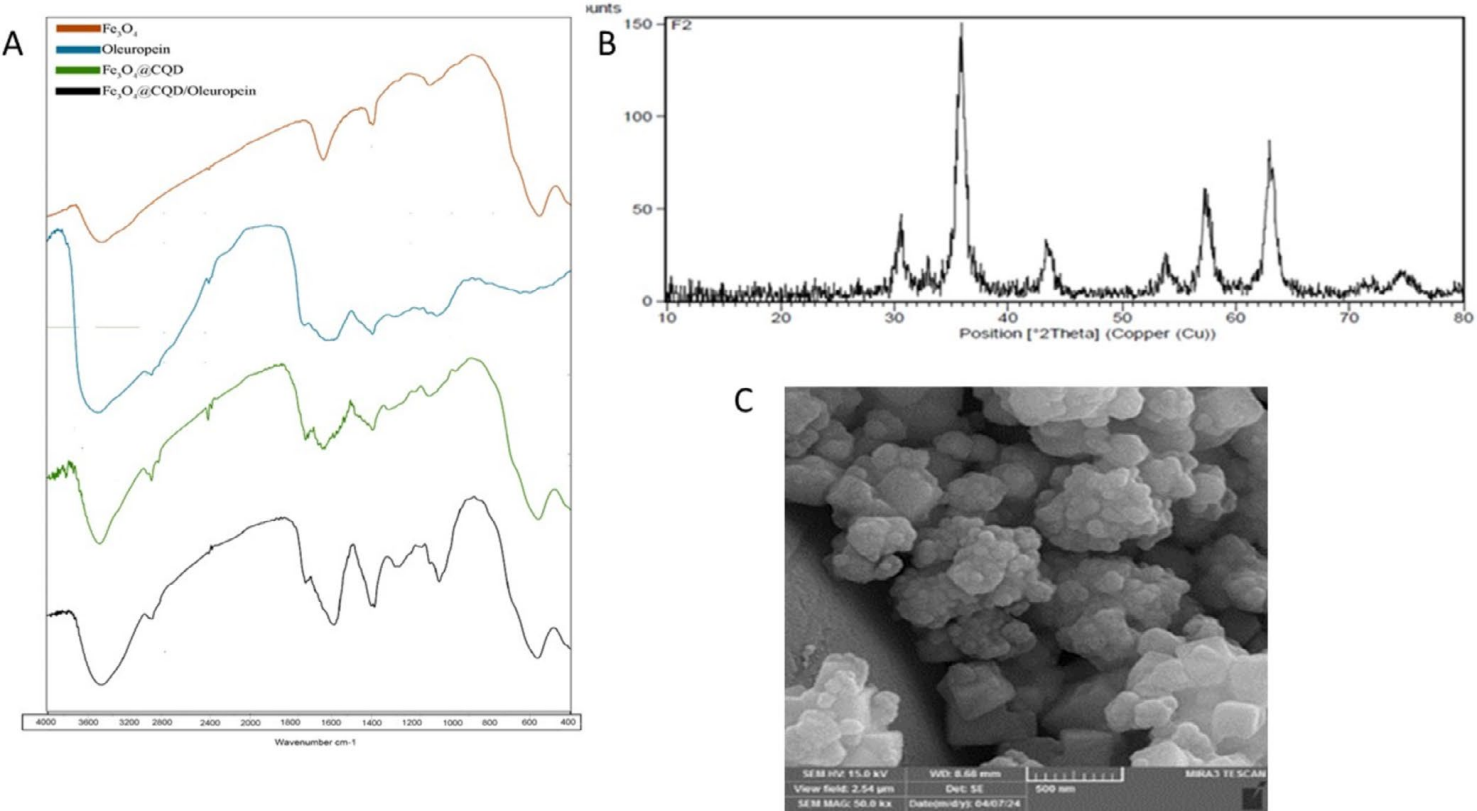
(A) FT‐IR spectra of Fe_3_O_4_, Oleuropein, Fe_3_O_4_@CQD, and Fe_3_O_4_@CQD/Oleuropein nanoparticles. (B) The X‐ray diffraction pattern of a sample of Fe_3_O_4_@CQD/Oleuropein. (C) The FE‐SEM image of Fe_3_O_4_@CQD/Oleuropein.

The FT‐IR spectra of Fe_3_O_4_, Ole, Fe_3_O_4_@CQD, and Fe_3_O_4_@CQD/Ole are shown in Figure 1. The functional groups of the synthesized nanoparticles were identified. The strong band at 3350–3500 cm^−1^ confirmed the O‐H stretching bond of the phenolic part of Ole and Fe_3_O_4_@CQD/Ole. The peaks at approximately 500–600 cm^−1^ correspond to Fe‐O bonding, confirming the presence of Fe_3_O_4_ in the structures of Fe_3_O_4_, Fe_3_O_4_@CQD, and Fe_3_O_4_@CQD/Ole. Fe_3_O_4_@CQD and Fe_3_O_4_@CQD/Ole exhibited a C=O stretching band at 1710 cm^−1^, signifying the presence of Fe_3_O_4_‐CQD. C–H stretching vibration signals were observed at 2920 cm^−1^. The characteristic vibration of the carbonyl groups in the Ole molecule was responsible for the signals observed at 1720 cm^−1^. The signal appeared at 1597 cm^−1^ as a stretching vibration and 1386 cm^−1^ as a bending vibration belonging to the functional groups C = C and O‐H, respectively. These results indicated the presence of Ole in Fe_3_O_4_@CQD/Ole (Figure 1A). The XRD pattern of Oleuropein attached to magnetite nanoparticles. All the signals in this pattern match the information from the standard XRD pattern of magnetite nanoparticles and confirm the crystallinity of the nanoparticles (Figure 1B). Fe_3_O_4_ nanoparticles with a spherical spinel structure showed signals at (311), (220), (440), (511), (422), and (400). The absence of (110) (2θ = 21.220) and (104) (2θ = 33.150) signals indicates that neither goethite (α‐FeOOH) nor hematite (α‐Fe_2_O_3_) phases were formed in the samples. Additionally, the binding of Oleuropein does not change the structure of Fe_3_O_4_@CQD; a broad peak is observed between 2θ = 12–28, which pertains to the overlap between Oleuropein and Fe3O4@CQD (Figure 1B). The morphology and size of the nanoparticles were determined using field‐emission scanning electron microscopy (FE‐SEM) technique. The scanning electron microscopy (SEM) image of the sample revealed a range of particle sizes, ranging from 30 to 80 nm. Therefore, the image illustrates that the particles of the sample ingredients exhibited a high degree of homogeneity and possessed a consistent, symmetrical, and uniform distribution (Figure 1C).

### 3.2 Characterization of Wharton's Jelly Derived Stem Cells and HFFF2 Cells

**FIGURE 2:**
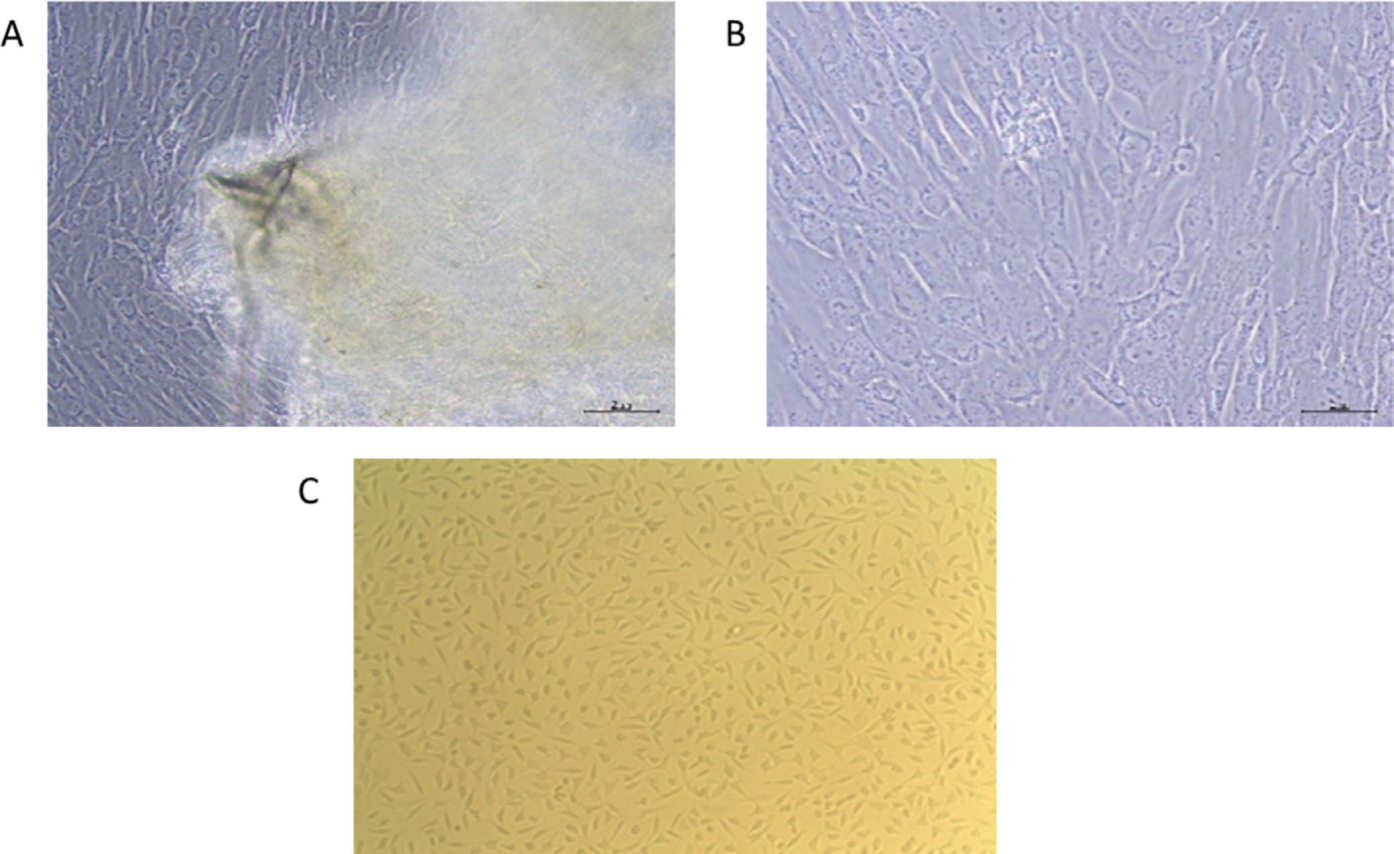
Morphological characteristics of Fibroblast‐like hUC‐MSCs. (A) Primary hUC‐MSCs on day 7 after culture. (B) Fibroblast‐like hUC‐MSCs at passage 3. (C) Morphological representation of normal human dermal fibroblasts (HFFF2).

Proliferating cells derived from human umbilical cord explants exhibited fibroblast‐like and spindle‐shaped morphologies (Figure 2A,B). The normal morphology of human dermal fibroblast cells (HFFF2) was observed using phase‐contrast microscopy (Figure 2C).

### 3.3 Viability Percentage (MTT Assay)

**FIGURE 3:**
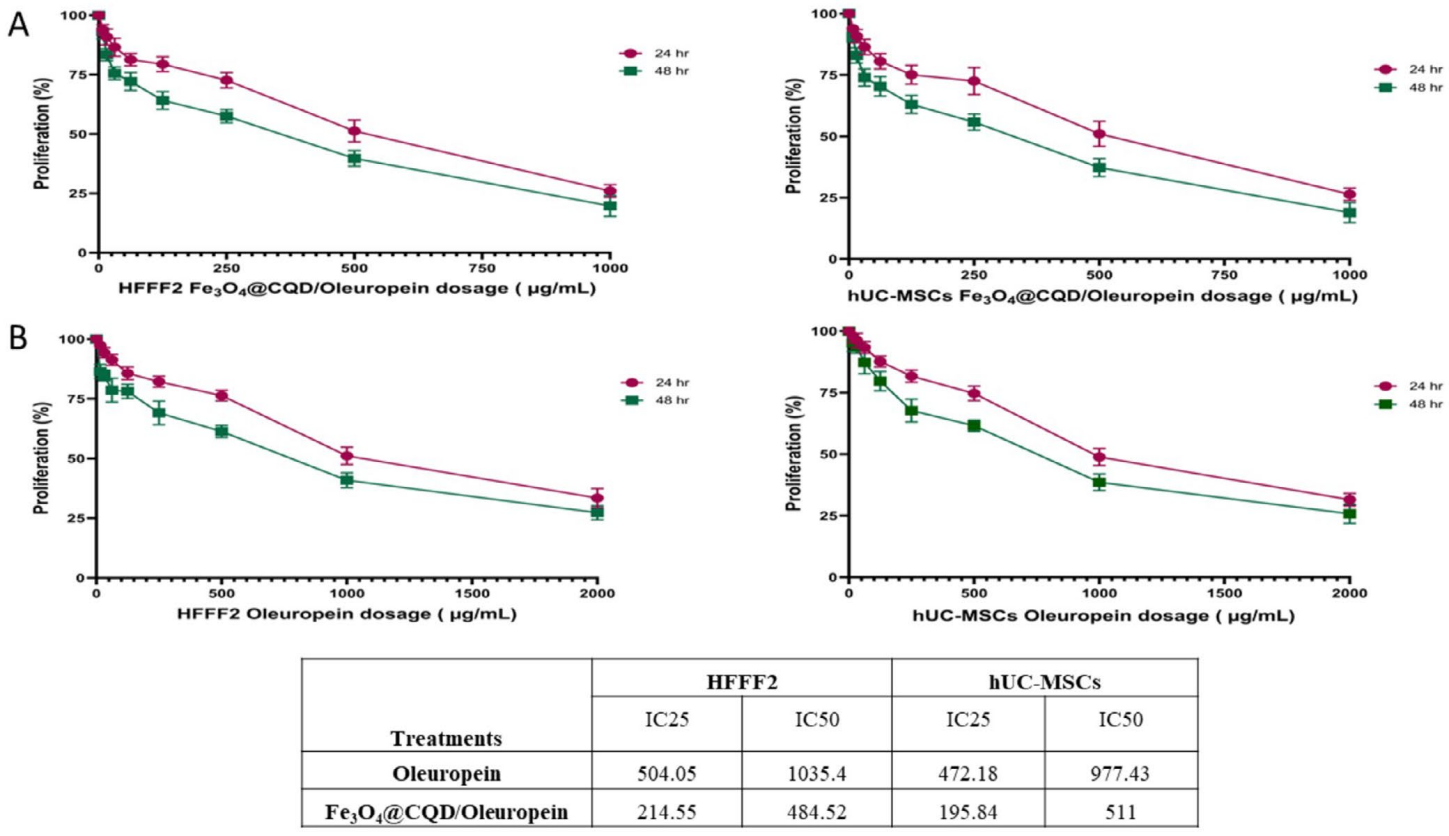
Cell viability. (A) HFFF2 and hUC‐MSCs cells treated with Fe@C/Ole in MTT assay. (B) HFFF2 and hUC‐MSCs cells treated with Ole in MTT assay.

Fe@C/Ole was evaluated as a conductive particle in terms of cell viability (Figure 3A), and the IC_50_ for the HFFF2 cells and hUC‐MSCs was 484.52 and 511 μg/mL, respectively. Cell treatment was performed using IC_25_ ≃ 250 μg/mL, although cell growth inhibition was also observed in this treatment.

The effects of different concentrations of Ole on the viability of HFFF2 and hUC‐MSCs were studied (Figure 3B). The amount of the IC_50_ was estimated at 1035.4 μg/mL for the HFFF2 cells and 977.43 μg/mL for hUC‐MSCs; cell treatment was performed utilizing IC_25_ ≃ 500 μg/mL for cell treatment as an effective concentration. In summary, the results showed that Ole has cell inhibition and toxicity in very high concentrations (IC_50_ ≃ 1000 μg/mL), whereas Fe@C/Ole has them in low concentrations (IC_25_ ≃ 500 μg/mL).

### 3.4 The Result of Exosome Characterization Assays

#### 3.4.1 Dynamic Light Scattering (DLS)

The exosomes isolated size from hUC‐MSCs treated with Fe@C/Ole, Ole, and the control group were 68.8 ± 4.82, 58.3 ± 4.49, and 41.3 ± 3.03 nm, respectively, and the exosomes isolated size from HFFF2 cells treated with Fe@C/Ole, Ole, and the control group were 67.3 ± 4.80, 42.6 ± 3.04, and 35.5 ± 2.82, respectively in the DLS analyses (Figures S1 and S2; Table 1). Z‐potential of the isolated exosomes was found to be −21.8 and −21.3 mV, which indicates the stability of exosomes in hUC‐MSCs treated with Ole and HFFF2 treated with Ole, respectively (Figure S3).

**TABLE 1:** DLS result to characterize size of isolated exosomes.

Treatments	Exosomes isolated size from HFFF2 cells (nm)	Exosomes isolated size from hUC‐MSCs (nm)
Control	35.5 ± 2.82	41.3 ± 3.03
Oleuropein	42.6 ± 3.04	58.3 ± 4.49
Fe_3_O_4_@CQD/Oleuropein	67.3 ± 4.80	68.8 ± 4.82

#### 3.4.2 Western Blot Analysis

**FIGURE 4:**
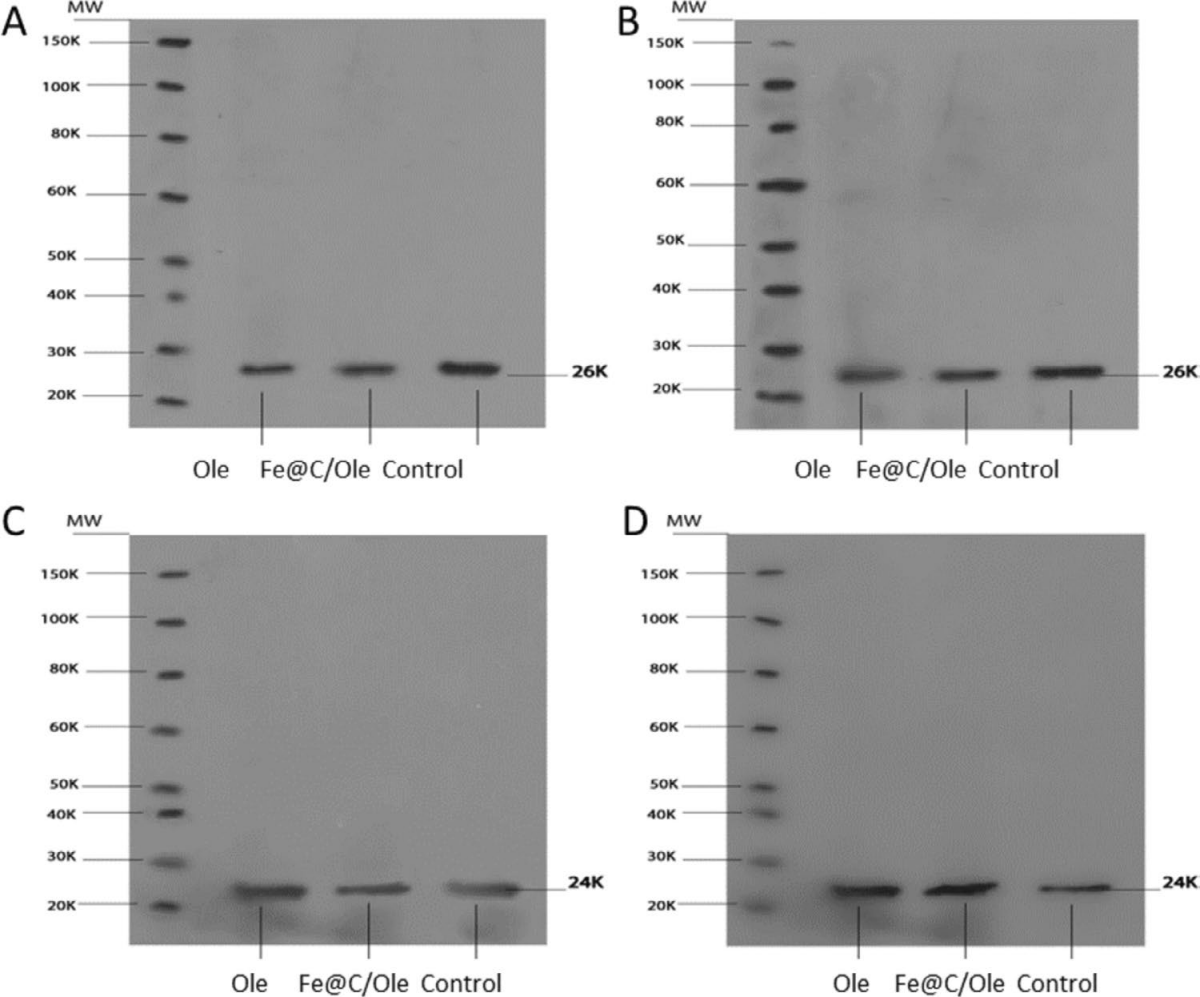
Western blot analysis. (A) shows complete blotting of CD63 (26 KDa) in hUC‐MSCs. (B) shows complete blotting of CD63 (26 KDa) in HFFF2 cells. (C) shows complete blotting of CD9 (24 KDa) in hUC‐MSCs. (D) shows complete blotting of CD9 (24 KDa) in HFFF2 cells.

In the western blot image, protein bands with a size of 26 kDa indicated exosome membrane protein CD63 (Figure 4A,B). The protein CD63 was present in the Fe@C/Ole, Ole, and control groups in both the hUC‐MSCs and HFFF2 cells. Furthermore, the presence of protein bands at 24 kDa implies the existence of the exosome membrane protein CD9, as depicted in Figure 4C,D. We detected CD9 protein in the hUC‐MSCs, HFFF2 cells, and control group.

### 3.5 In Silico Analysis of microRNAs Related to Studied Genes

In silico analysis was performed to determine and confirm the selection of microRNAs associated with the studied genes, as detailed in Table 2. Specifically, for the *IGF1* gene, *hsa‐miR‐29b‐3p* and *hsa‐miR‐34a‐5p* were identified. The *IGF1R* gene was associated with *hsa‐miR‐29b‐3p*, *hsa‐miR‐34a‐5p*, *hsa‐let‐7d‐5p*, and *hsa‐let‐7e‐5p*. The *EGF* gene was linked to *hsa‐miR‐34a‐5p*. For *COL1A1*, *hsa‐miR‐29b‐3p*, *hsa‐miR‐34a‐5p*, and *hsa‐let‐7d‐5p* were confirmed. Lastly, *hsa‐miR‐29b‐3p* was associated with the *ELN* gene (Table 2).

**TABLE 2:** The list of selected microRNAs related to studied genes.

Target genes	microRNAs	References
IGF1	hsa‐miR‐29b‐3p	[39, 40]
	hsa‐miR‐34a‐5p	
	hsa‐miR‐29b‐3p	
IGF1R	hsa‐miR‐34a‐5p	[41, 42, 43]
	hsa‐let‐7d‐5p	
	hsa‐let‐7e‐5p	
EGF	hsa‐miR‐34a‐5p	[44]
	hsa‐miR‐29b‐3p	
COL1A1	hsa‐miR‐34a‐5p	[45, 46, 47]
	hsa‐let‐7d‐5p	
ELN	hsa‐miR‐29b‐3p	[48]

### 3.6 Gene and microRNA Expression

#### 3.6.1 The Relative Gene Expression Treated With Fe@C/Ole and Ole

In the ANOVA test, significant differences were observed between the treatments (Fe@C/Ole, Ole, and control) in the HFFF2 cells, in terms of the relative expression of *IGF1*, *IGF1R*, *COL1A1*, *ELN*, and *EGF* genes (*p* ≤ 0.05). Also, in the hUC‐MSCs, there was a significant difference in the relative expression (*p* ≤ 0.05) of *IGF1*, *IGF1R*, *COL1A*, and *ELN* genes between the treatments. In contrast to other genes, no significant difference was observed in the relative gene expression of EGF in the hUC‐MSCs between the treatments (Table S3).

**FIGURE 5:**
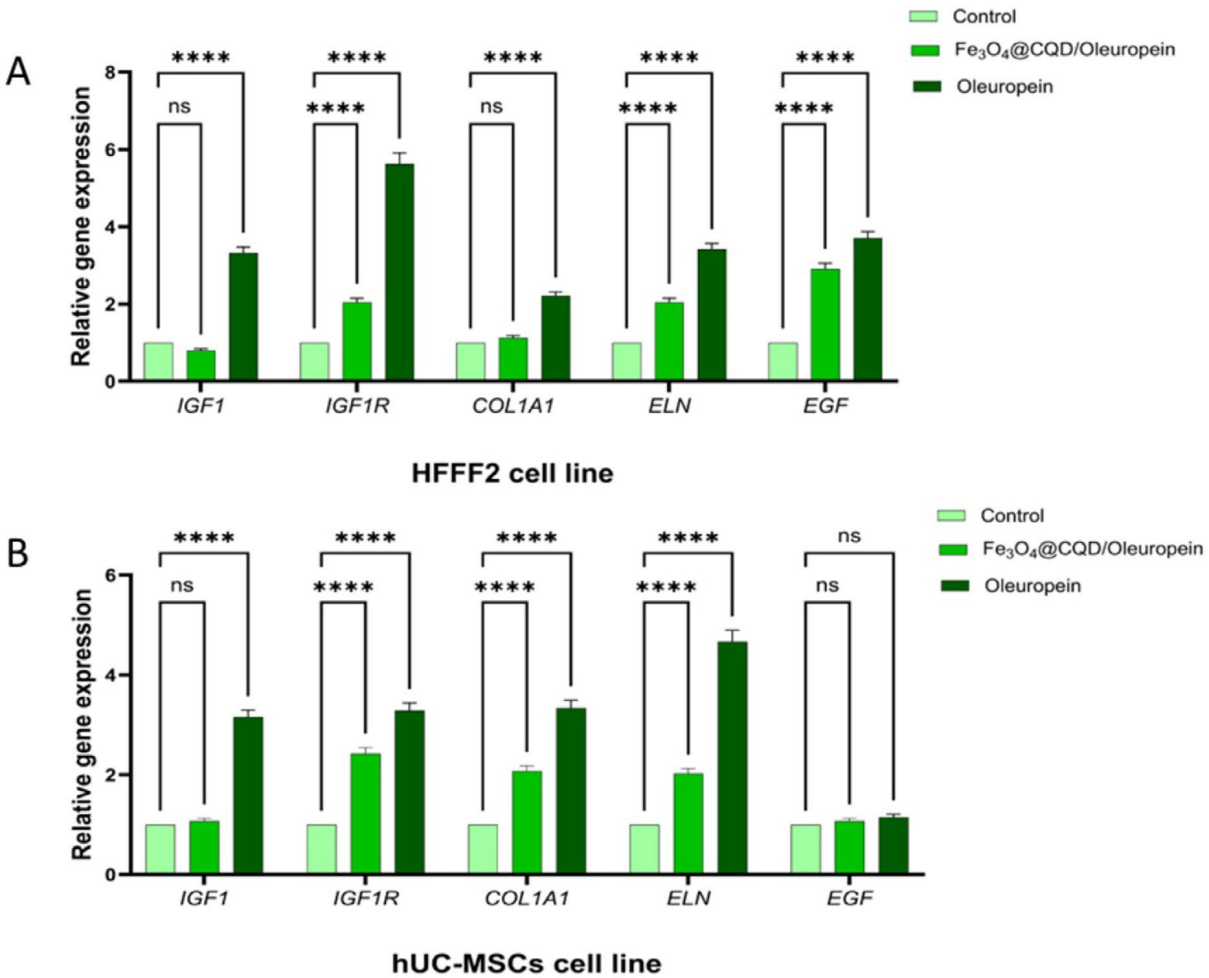
Relative expression of IGF1, IGF1R, COL1A1, ELN, and EGF genes in treatment with Fe@C/Ole, Ole, and the control. (A) HFFF2 cells. (B) hUC‐MSCs. The results are illustrated as mean ± SD. Significant differences between treatments compared to control were calculated based on (ns) nonsignificant, (*) *p* ≤ 0.05, (**) *p* ≤ 0.01, (*** or more) *p* ≤ 0.001.

The HFFF2 cells exhibited significant upregulation in the expression of the *IGF1* (fold change: 3.14), *IGF1R* (5.3), *COL1A1* (2.11), *ELN* (3.17), and *EGF* (3.5) genes in response to Ole treatment compared to the control group. Additionally, Fe@C/Ole treatment upregulated the expression of the *IGF1R* (2.03), *ELN* (1.94), and *EGF* (2.76) genes compared with the control group; however, there were no significant changes in the *IGF1* and *COL1A1* genes in the Fe@C/Ole group compared with the control group (Figure 5A). This trend of gene expression changes was similar in hUC‐MSCs, although, as mentioned, the EGF gene was not significant, and, unlike the HFFF2 cells, the *COL1A1* gene was upregulated in the Fe@C/Ole treatment (Figure 5B).

#### 3.6.2 The Relative Gene Expression in Exosome‐Treated HFFF2 Cells

According to the qRT‐PCR data and analysis of variance, there was a significant difference between HFFF2‐derived exosomes and hUC‐MSC‐derived exosomes treated with Fe@C/Ole, Ole, and control treatments in terms of the relative expression of *IGF1*, *IGF1R*, *COL1A1*, *ELN*, and *EGF* genes (*p ≤ 0.05*) in HFFF2 cells (Table S4).

**FIGURE 6:**
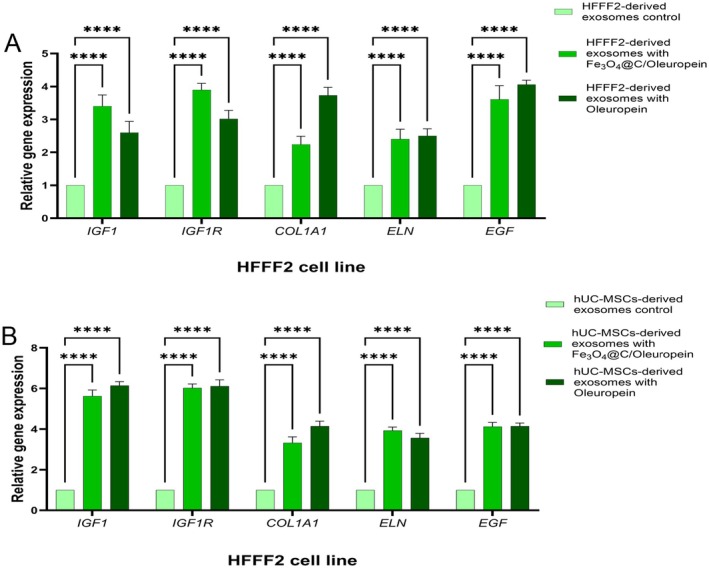
Relative expression of IGF1, IGF1R, COL1A1, ELN, and EGF genes. (A) HFFF2 cells treated with HFFF2‐derived exosomes with Fe@C/Ole, Ole, and control. (B) HFFF2 cells treated with hUC‐MSCs‐derived exosomes with Fe@C/Ole, Ole, and control. The results are illustrated as mean ± SD. Significant differences between treatments compared to control were calculated based on (ns) nonsignificant, (*) *p* ≤ 0.05, (**) *p* ≤ 0.01, (*** or more) *p* ≤ 0.001.

The findings indicated that the relative expression levels of *IGF1* (2.6, 6.14), *IGF1R* (3.02, 6.11), *COL1A1*(3.73, 4.25), *ELN* (2.5, 3.25), and *EGF* (4.06, 4.14) were strongly upregulated in the HFFF2 cells treated with HFFF2‐derived and hUC‐MSC‐derived exosomes with Ole compared to the control group, respectively (Figure 6A,B). Also, the results showed that the relative expression levels of *IGF1* (3.4, 5.62), *IGF1R* (3.9, 6.02), *COL1A1* (2.24, 3.32), *ELN* (2.4, 3.4), and *EGF* (3.61, 4.12) were significantly upregulated in the HFFF2 cells after the treatment of HFFF2‐derived and hUC‐MSC‐derived exosomes with Fe@C/Ole compared to the control group, respectively (Figure 6A,B). Overall, the effects of HFFF2‐derived and hUC‐MSC‐derived exosomes were similar; however, the effect of exosome treatment on gene expression was more significant than that of direct treatment with Fe@C/Ole and Ole compared to the control group.

#### 3.6.3 The Relative microRNA Expression in the Content of Exosomes From Fe@C/Ole and Ole‐Treated Cells

**FIGURE 7:**
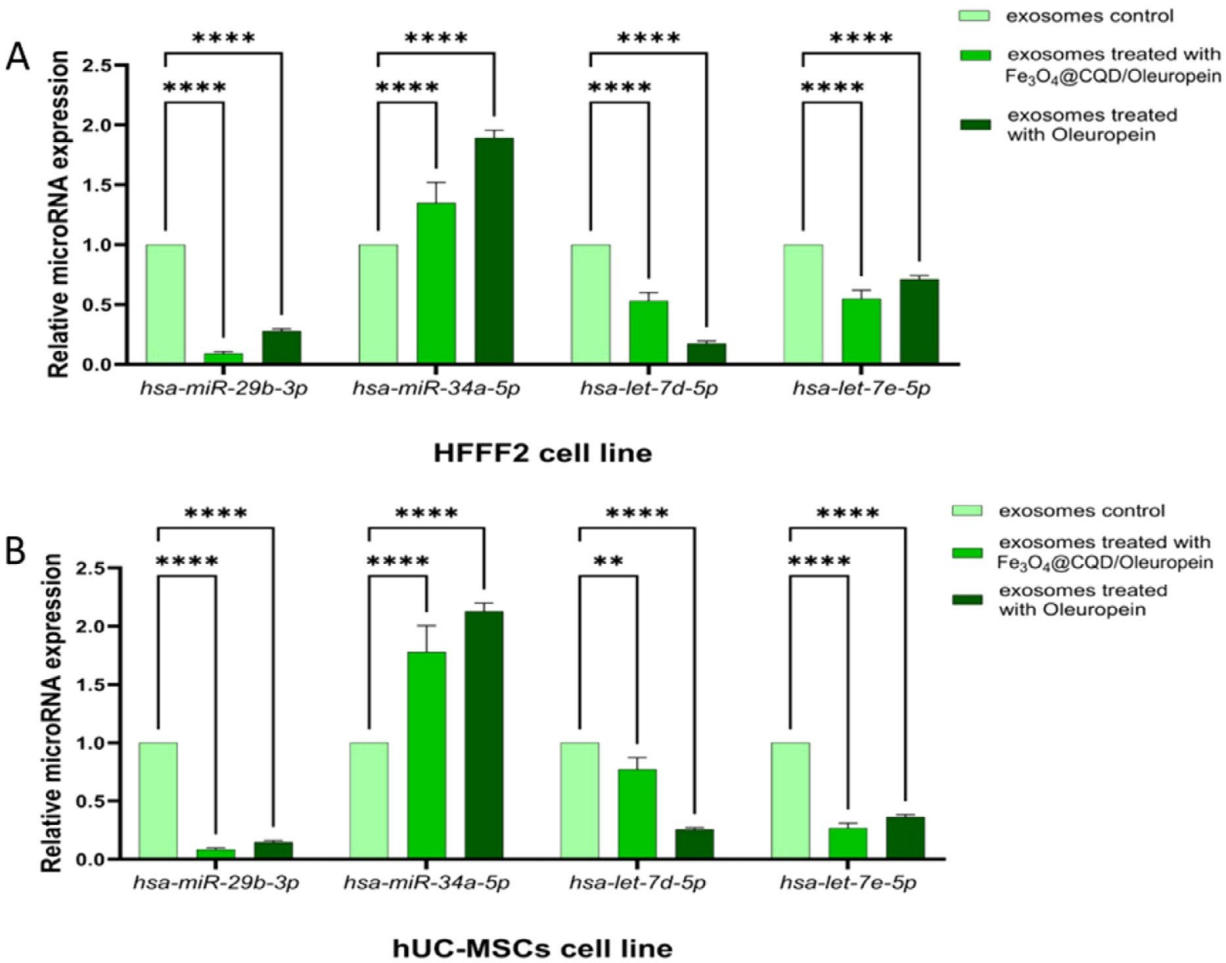
Relative expression of hsa‐miR‐29b‐3p, hsa‐miR‐29c‐3p, hsa‐miR‐9‐5p, hsa‐miR‐155‐5p, hsa‐miR‐34a‐5p, hsa‐let‐7d‐5p, and hsa‐let‐7e‐5p microRNAs treated with Fe@C/Ole and Ole compared to the control. (A) HFFF2‐derived exosomes. (B) hUC‐MSCs‐derived exosomes. The results are illustrated as mean ± SD. Significant differences between treatments compared to control were calculated based on (ns) nonsignificant, (*) *p* ≤ 0.05, (**) *p* ≤ 0.01, (*** or more) *p* ≤ 0.001.

In the ANOVA, significant differences were observed between the treatments (Table S5). The results showed that *hsa‐miR‐29b‐3p*, *hsa‐let‐7d‐5p*, and *hsa‐let‐7e‐5p* microRNAs were significantly downregulated, whereas *miR‐34a‐5p* was upregulated in HFFF2‐derived exosome content, as well as in hUC‐MSC‐derived exosome content treated with Fe@C/Ole and Ole compared to the control (Figure 7A,B).

#### 3.6.4 The Relative Expression of microRNAs in HFFF 2 Cells Treated With Cell‐Derived Exosomes

**FIGURE 8:**
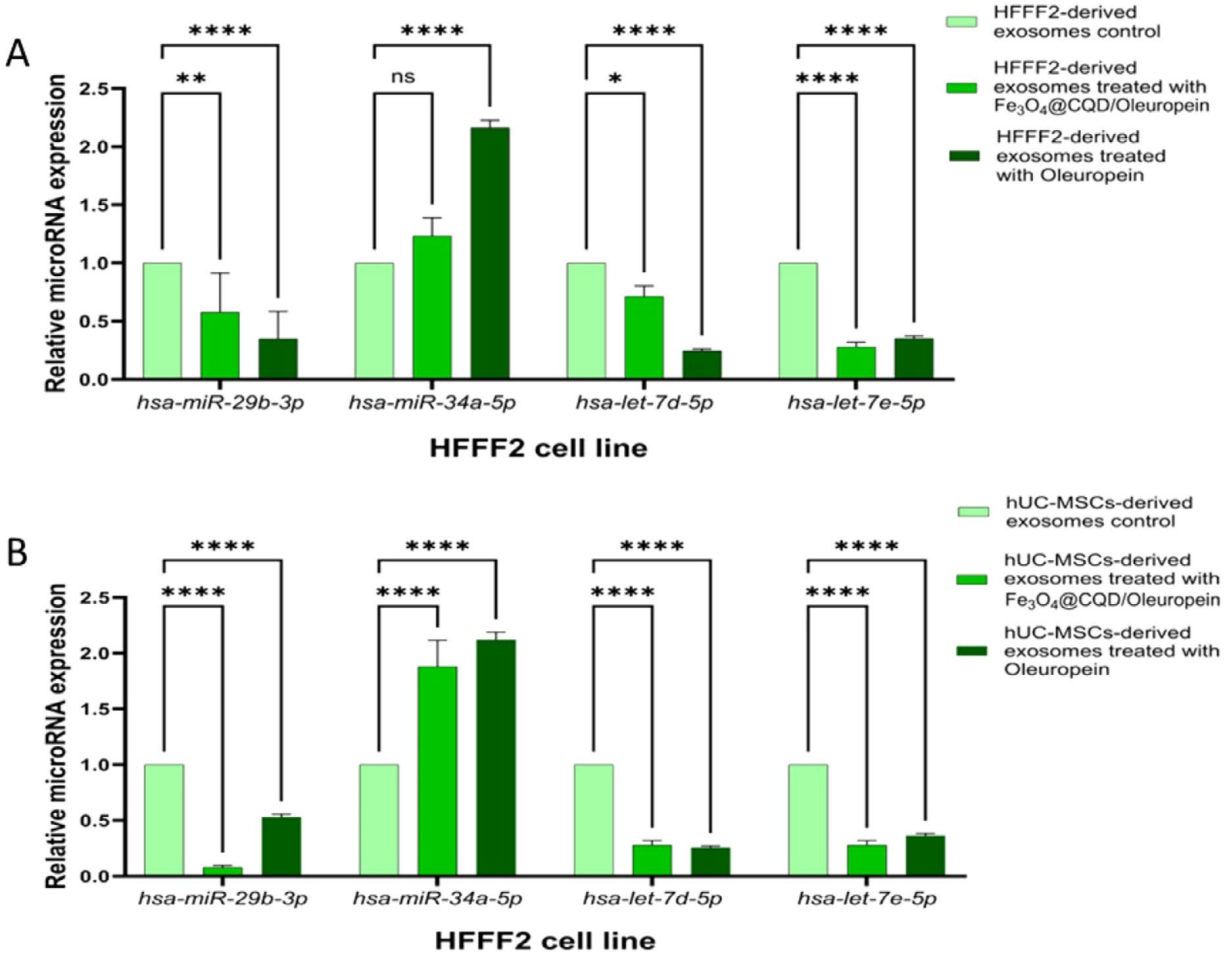
Relative expression of hsa‐miR‐29b‐3p, hsa‐miR‐29c‐3p, hsa‐miR‐9‐5p, hsa‐miR‐155‐5p, hsa‐miR‐34a‐5p, hsa‐let‐7d‐5p, and hsa‐let‐7e‐5p microRNAs in HFFF2 cells. (A) treated with HFFF2‐derived exosomes. (B) treated with hUC‐MSCs‐derived exosomes. The results are illustrated as mean ± SD. Significant differences between treatments compared to control were calculated based on (ns) nonsignificant, (*) *p* ≤ 0.05, (**) *p* ≤ 0.01, (*** or more) *p* ≤ 0.001.

In the ANOVA test, significant changes were observed between the treatments (Table S6). The present study evaluated the amounts of different microRNAs in HFFF2 cells treated with HFFF2‐derived exosomes and in hUC‐MSC‐derived exosomes in comparison to the control. The results indicated that the *hsa‐miR‐29b‐3p*, *hsa‐let‐7d‐5p*, and *hsa‐let‐7e‐5p* microRNAs were downregulated in HFFF2 cells between the treatments. In contrast, *hsa‐miR‐34a‐5p* was upregulated between treatments in HFFF2 cells (Figure 8A,B).

#### 3.6.5 Expression Correlation of microRNAs

Our investigation demonstrated a statistically significant correlation between the expression levels of specific microRNAs in modified exosomal content and the associated alterations in HFFF2 cells treated with these exosomes. This finding supports the concept that exosomal miRNAs are conveyed to recipient cells and influence their gene expression.

The study examined expression correlation of several microRNAs, including *hsa‐miR‐29b‐3p*, *hsa‐miR‐34a‐5p*, *hsa‐let‐7d‐5p*, and *hsa‐let‐7e‐5p*, in HFFF2‐derived exosomes treated with Fe@C/Ole and Ole compared to exosomes of HFFF2 cells that were treated with exosomes derived from these cells. Significant positive correlations were consistently observed for these microRNAs in exosomes and exosomes of HFFF2 cells, indicating a robust influence on HFFF2 cellular responses. These results highlight the critical role of exosomes in intercellular communication and the regulation of gene expression within recipient cells (Table S7).

### 3.7 Results of Exosome Toxicity Investigation

**FIGURE 9:**
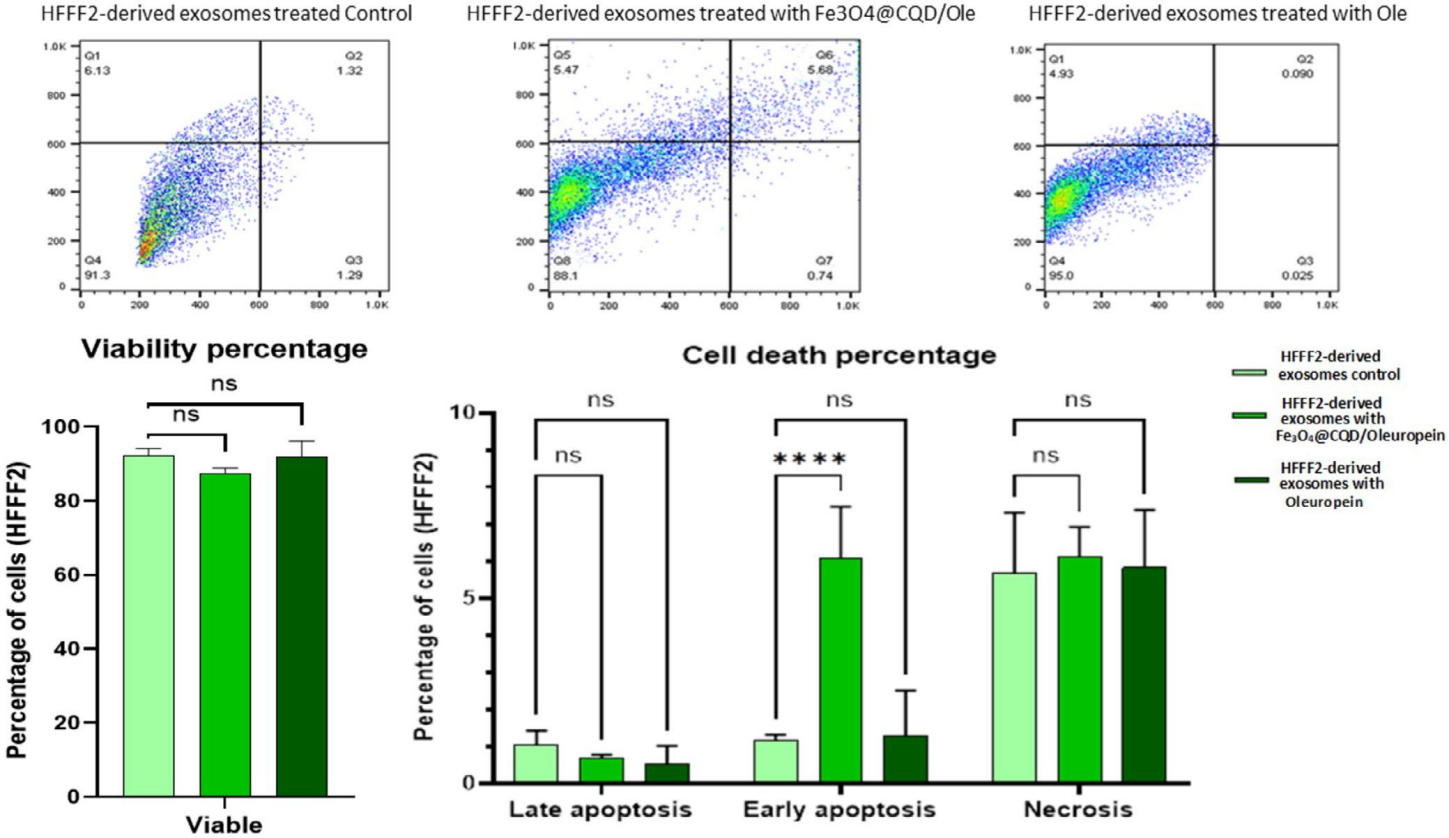
Flow Cytometry showed no significant difference between the HFFF2‐derived exosomes treated with Fe@C/Ole, Ole, and the control in viability, late apoptosis, and necrosis, but in early apoptosis, it was seen that the HFFF2‐derived exosomes treated with Fe@C/Olesignificantly increased.

The Flow Cytometry analysis results showed no significant variations in the viability percentage between HFFF2‐derived exosomes treated with Fe@C/Ole, Ole, or the control in the HFFF2 cells (Figure 9). Flow Cytometry analysis of cell death data, including late apoptosis and necrosis, revealed no significant differences between exosome treatments. However, the HFFF2‐derived exosomes treated with Fe@C/Ole significantly increased early apoptosis (*p* ≤ 0.05); on the other hand, there were no significant differences (*p* ≤ 0.05) in early apoptosis between the HFFF2‐derived exosomes treated with Ole and the control in the HFFF2 cells (Figure 9).

**FIGURE 10:**
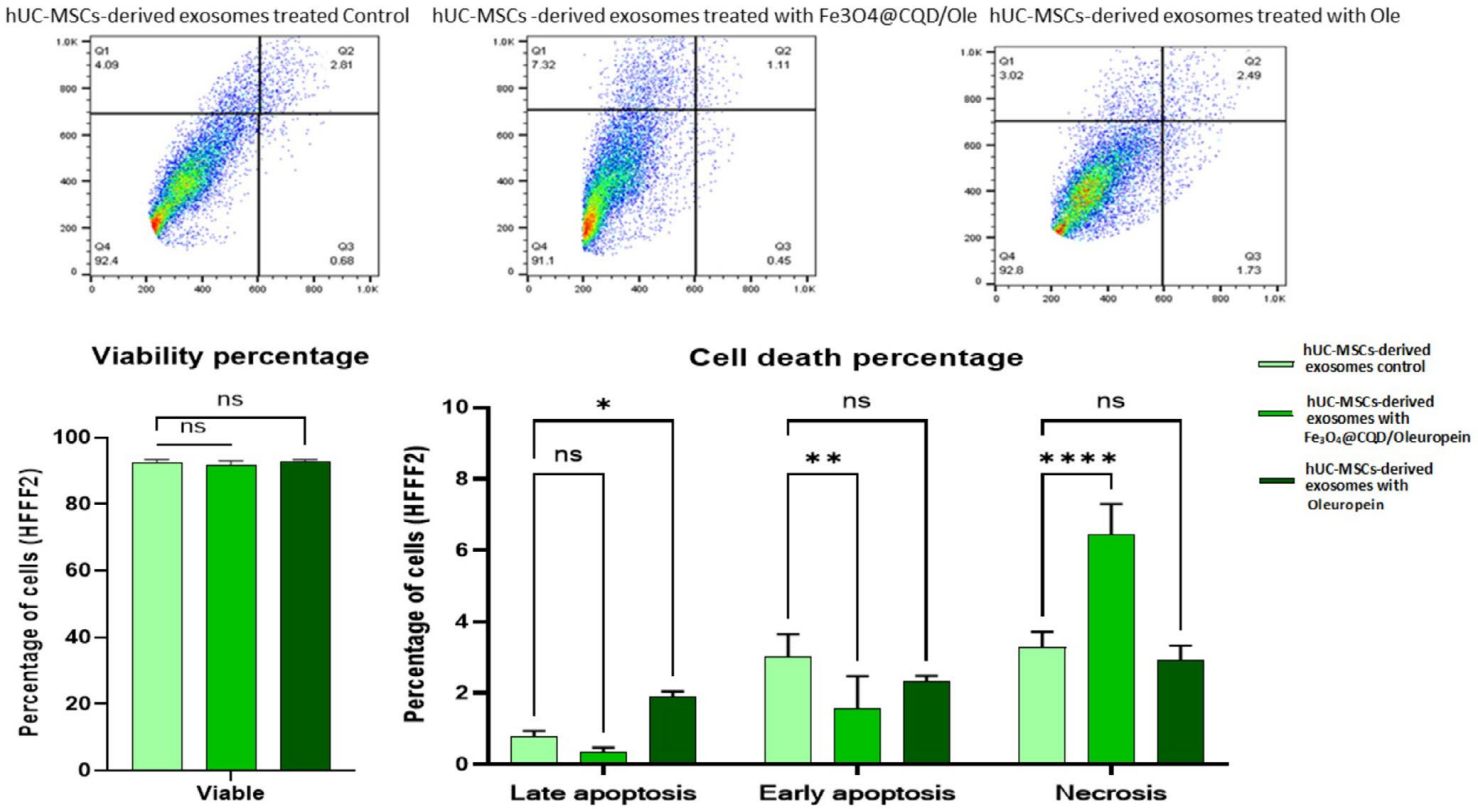
Flow Cytometry showed no significant difference between the hUC‐MSCs‐derived exosomes treated with Fe@C/Ole, Ole, and the control in viability; however, late apoptosis was significantly increased in the hUC‐MSCs‐derived exosomes treated with Ole, early apoptosis was significantly reduced in the hUC‐MSCs‐derived exosomes treated with Fe@C/Ole, and necrosis was significantly increased in the hUC‐MSCs‐derived exosomes treated with Fe@C/Ole.

The Flow Cytometry experiment demonstrated no significant differences in the viability percentage between hUC‐MSC‐derived exosomes treated with Fe@C/Ole, Ole, and the control group in the HFFF2 cells. In addition, Flow Cytometry analysis of cell death data, including early apoptosis and necrosis, revealed no significant differences in hUC‐MSC‐derived exosomes treated with Ole compared with the control. However, there was a significant increase in late apoptosis in the hUC‐MSC‐derived exosomes treated with Ole compared to the control (*p ≤* 0.05); on the other hand, there were significant differences in early apoptosis and necrosis in the hUC‐MSC‐derived exosomes treated with Fe@C/Ole compared to the control in the HFFF2 cells. Also, in late apoptosis, there were no significant differences in the hUC‐MSC‐derived exosomes treated with Fe@C/Ole compared to the control in the HFFF2 cells (Figure 10).

## 4 Discussion

Previous research has revealed that the IGF1, EGFR, MAPK, and PI3K signaling pathways are involved in skin rejuvenation [34, 35, 36, 37]. For these signaling pathways, we selected to investigate growth factors and their receptors, including the *IGF1*, *IGF1R*, and *EGF* genes, as well as the *COL1A1* and *ELN* genes, which are primarily involved in skin renewal. Conversely, microRNAs modulate gene expression in the skin aging process. Research has identified multiple microRNAs linked to skin aging, which also target our chosen genes, notably the *hsa‐miR‐29*, *hsa‐miR‐34*, and *hsa‐let‐7* families [49]. Research has shown that *IGF1* is regulated by *hsa‐miR‐29b‐3p* [39] and *hsa‐miR‐34a‐5p* [40]; *IGF1R* is targeted by *hsa‐miR‐29b‐3p* [41], *hsa‐miR‐34a‐5p* [42], *hsa‐let‐7d‐5p* [43], and *hsa‐let‐7e‐5p* [43]; *EGF* is regulated by *hsa‐miR‐34a‐5p* [44]; *COL1A1* is targeted by *hsa‐miR‐29b‐3p* [45], *hsa‐miR‐34a‐5p* [46], and *hsa‐let‐7d‐5p* [47]; and *ELN* is regulated by *hsa‐miR‐29b‐3p* [48].

Aging causes an increase in *MMPs* and a reduction in Collagen (*COL1A1*) and Elastin (*ELN*) levels [50, 51]. Thus, enhancing the production of Collagen (*COL1A1*) and Elastin (*ELN*) can effectively mitigate the effects of aging and promote rejuvenation [51, 52]. This study revealed that Ole and Fe@C/Ole (slow release) treatments increased *COL1A1* and *ELN* mRNA levels in HFFF2 cells and hUC‐MSCs that support the hypothesis of skin rejuvenation through treatments, as *MMP9* (nonreported) has the ability to degrade collagen and elastin. Results were anticipated given the proven efficacy of Ole in many signaling pathways, including IGF1, EGFR, MAPK, and PI3K [13, 53]. The effect of Ole can be attributed to its antioxidant effects, as well as its influence on the expression of growth factors such as *IGF1* and *EGF* [10, 54, 55]. Generally, in our study, Ole treatment upregulated the expression of *IGF1*, *EGF*, and *IGF1R*, but *IGF1* was not significant in Fe@C/Ole treatment (Figures 5 and 6). To date, reports have shown that antioxidant treatments, such as Ole, quercetin, and resveratrol, increase the expression of *IGF1*, *EGF*, and *IGF1R* genes [56, 57].

**FIGURE 11:**
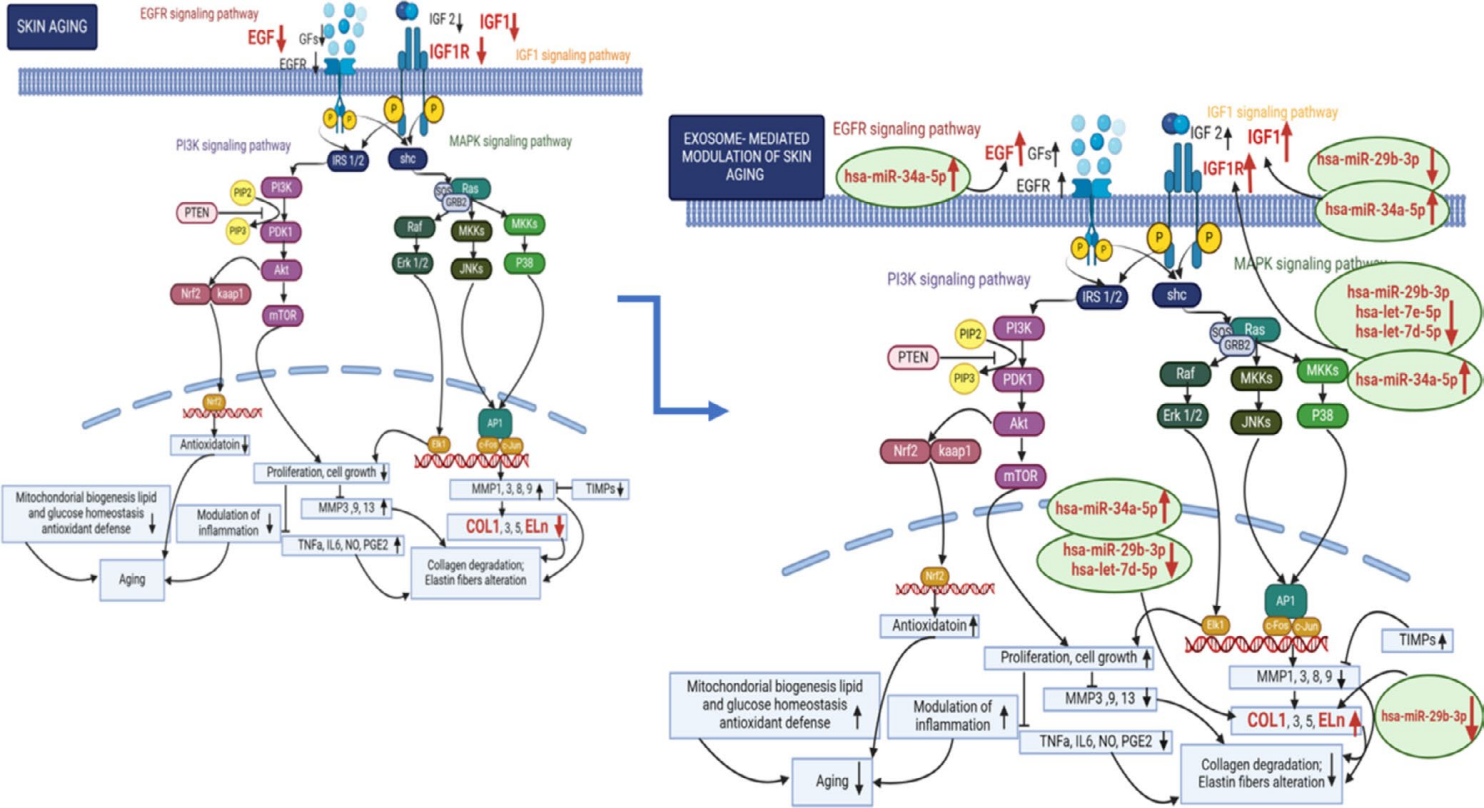
Modulation of skin aging through study modified exosomal contents on the IGF1, EGFR, MAPK, and PI3K signaling pathways related to skin aging.

The nanocomposite structure can improve Ole's stability and activity by preventing degradation in biological fluids. This is crucial for maintaining therapeutic levels during treatment [58]. Ole's effect on enhancing these growth factors and their receptors has been examined by targeting the MAPK signaling pathway [53, 59], which supports the rejuvenation process by inhibiting *MMPs* and promoting the production of *COL1A1* and *ELN* (Figure 11). Furthermore, previous studies have highlighted Ole's ability to activate the PI3K signaling pathway and its significant antioxidant effects [60]. This pathway is known for its antioxidant properties; by increasing the expression of antioxidant cascades within the cell's nucleus and mitochondria, it actively inhibits the aging process [61]. Additionally, reducing *MMP* activity and blocking inflammatory pathways—such as those involving *prostaglandin E2* (*PGE2*), *tumor necrosis factor* (*TNF*), *nitric oxide* (*NO*), and *interleukin 6* (*IL‐6*)—promotes the synthesis of *collagen* (*COL1*) and *elastin* (*ELN*) (Figure 11).

In fact, our investigation involved treating HFFF2 cells with exosomes derived from both HFFF2 cells and hUC‐MSCs, all of which had been pre‐treated with either Ole or Fe@C/Ole. This approach led to an observable increase in the expression of several key genes: *IGF1*, *IGF1R*, *COL1A1*, *ELN*, and *EGF* (Figure 11). This outcome aligns generally with findings from direct Ole therapy, with the notable exception of the *EGF* gene's response in hUC‐MSCs. Interestingly, whereas direct treatment of HFFF2 cells with Fe@C/Ole did not significantly alter the expression of *IGF1* and *COL1A1* and similarly for *EGF* and *IGF1* in hUC‐MSCs, a significant upregulation was observed when these cells were instead treated with exosomes originating from HFFF2 cells and hUC‐MSCs that had undergone Fe@C/Ole pre‐treatment. This suggests that the reduced toxicity of Fe@C/Ole when delivered via exosomes appears to have notably increased gene expression within the exosomes themselves, a significant improvement compared to direct application. Utilizing modified exosomal content offers distinct benefits over conventional treatments. Primarily, these modified exosomes do not trigger the body's antibody and antigen systems, thus avoiding an immune response. Secondly, their internal components are directly influenced by the initial treatment, as these exosomes are typically derived from living cells [22, 62]. Furthermore, both Ole therapy and Fe@C/Ole application substantially altered the makeup of the exosomes, leading to observable changes in their modified content. Exosomes also exhibit remarkable anti‐inflammatory properties, which contribute to accelerated healing, a reduced risk of EGF complications such as post‐inflammatory hyperpigmentation and scarring, and improved results in photo rejuvenation by enhancing collagenases and promoting the proliferation of dermal fibroblasts [63, 64].

On the other hand, this investigation focused on microRNAs linked to genes involved in the IGF1, EGFR, MAPK, and PI3K signaling pathways. We assessed how Ole and Fe@C/Ole treatments affected the levels of exosomal microRNAs in HFFF2 cells and hUC‐MSCs, given their known association with growth factors and genes critical for skin rejuvenation. The findings indicated that a 24‐h treatment influenced the expression levels of *hsa‐miR‐29b‐3p*, *hsa‐miR‐34a‐5p*, *hsa‐let‐7d‐5p*, and *hsa‐let‐7e‐5p* microRNAs in exosomes. The changes in Ole and Fe@C/Ole treatments resulted in the downregulation of *hsa‐miR‐29b‐3p*, *hsa‐let‐7d‐5p*, and *hsa‐let‐7e‐5p* microRNAs in exosomes derived from HFFF2 cells and hUC‐MSCs. Downregulation of *hsa‐miR‐29b‐3p*, *hsa‐let‐7d‐5p*, and *hsa‐let‐7e‐5p* microRNAs as interfering RNAs justified the upregulation of *IGF1*, *IGF1R*, *COL1A1*, *ELN*, and *EGF* gene expression (Table 2). These observations were corroborated by the expression of the aforementioned genes within HFFF2 cells, subsequent to their treatment with HFFF2‐derived exosomes that had undergone pre‐treatment with Ole and Fe@C/Ole (Figure 6). This research demonstrates a clear alignment between the microRNA levels found within the exosomes and those present in the HFFF2 recipient cells influenced by these exosomes. Consequently, modified exosomal content, produced through Ole and Fe@C/Ole therapy, emerges as a practical substitute for directly treating cells. These tiny vesicles hold considerable benefits when compared to traditional topical facial applications, positioning them as a more effective therapeutic choice [65, 66].

Unexpectedly, overexpression of *hsa‐miR‐34a‐5p* in exosomes treated with Fe@C/Ole and Ole and also exosomes of HFFF2 cells that were treated with exosomes derived from these cells indicates a complex role in skin aging responses. It enhances skin repair by promoting cell turnover and healthier epidermal layers, regulates oncogene‐suppressing pathways to prevent skin deterioration, and induces apoptosis to clear damaged cells, supporting new cell generation. Additionally, it modulates genes vital for tissue repair, boosting collagen and elastin synthesis for improved skin youthfulness. These effects collectively enhance skin self‐renewal, underscoring its potential in antiaging research [67, 68, 69, 70].

This study provides novel insights into the regenerative properties of modified exosomal contents, indicating that Ole and Fe@C/Ole in exosome‐based therapies hold great promise for skin rejuvenation. In addition, this study offers important insights into the effects of Ole and Fe@C/Ole on cellular mechanisms, and it is important to understand that the experiments were performed in vitro using cell lines. Applying these findings to in vivo conditions, such as human skin, could require more studies. The intricacies of the human body, including factors such as skin thickness and blood circulation, may affect the efficacy and potential side effects of Ole or Fe@C/Ole‐based treatments.

## 5 Conclusion

The research findings indicate that the use of modified exosomal content with Ole and Fe@C/Ole (slow release) treatment is a more effective method for transferring substances and biomolecules that are effective in the molecular mechanisms of skin rejuvenation than the use of effective substance treatment alone in vitro. This method can ultimately increase collagen and elastin gene expression while reducing interfering microRNAs in their respective genes.

## Author Contributions

**Naeimeh Safavizadeh:** data curation, funding acquisition, investigation, methodology, software, visualization, writing – original draft. **Zahra Noormohammadi:** supervision, validation, writing – review and editing. **Mohammad Zaefizadeh:** conceptualization, formal analysis, methodology, project administration, resources, supervision, validation, writing – review and editing. **Kazem Nejati‐Koshki:** supervision, writing – review and editing.

## Ethics Statement

The study was conducted according to the Declaration of Helsinki, and the protocol was approved by the Research Ethics Committee of Azad University, Ardabil Branch, Iran, with ethics code IR.IAU.ARDABIL.REC.1402.054. The people's informed written consent was obtained before participating.

## Consent

All authors have read and approved the manuscript for publication and consent to its publication in *Journal of Cosmetic Dermatology*.

## Conflicts of Interest

The authors declare no conflicts of interest.

## Supporting information


**Figure S1.** DLS to characterize the size of exosomes in hUC‐MSCs. (a) Fe3O4@CQD/Ole, (b) Ole, (c) Control group.
**Figure S2.** DLS to characterize size of exosomes in HFFF2 cell line. (a) Fe3O4@CQD/Ole, (b) Ole, (c) Control group.
**Figure S3.** Graph of zeta potential under ole treatment in (a) hUC‐MSCs and (b) HFFF2 cell line.
**Table S1.** Gene primer sequences used for qRT‐PCR.
**Table S2.** microRNA primer sequences used for qRT‐PCR.
**Table S3.** The result of ANOVA test (MS) for Fe@C/Ole and Ole treatments.
**Table S4.** The result of ANOVA test (MS) for exosomes treatments.
**Table S5.** The result of ANOVA test (MS) in exosomes.
**Table S6.** The result of ANOVA test (MS) HFFF2 cell line treated with exosomes.
**Table S7.** Correlation Analysis of MicroRNA Expression in HFFF2‐derived exosomes treated with Fe@C/Ole and Ole compared to exosomes of HFFF2 cells that were treated with exosomes derived from these cells.

## Data Availability

The data that supports the findings of this study are available in the Supporting Information of this article.
