# Supplementary material for: Enhancing Skin Rejuvenation: Using of Engineered Exosome Content Treated With Oleuropein and Fe3O4 @CQD/Oleuropein
Source: J Cosmet Dermatol. 2025 Jul 14;24(7):e70351. doi: 10.1111/jocd.70351 (PMC12257441; doi:10.1111/jocd.70351)
Supplement: Supplementary file 1 — Figure S1. DLS to characterize the size of exosomes in hUC‐MSCs. (a) Fe3O4@CQD/Ole, (b) Ole, (c) Control group. Figure S2. DLS to characterize size of exosomes in HFFF2 cell line. (a) Fe3O4@CQD/Ole, (b) Ole, (c) Control group. Figure S3. Graph of zeta potential under ole treatment in (a) hUC‐MSCs and (b) HFFF2 cell line. Table S1. Gene primer sequences used for qRT‐PCR. Table S2. microRNA primer sequences used for qRT‐PCR. Table S3. The result of ANOVA test (MS) for Fe@C/Ole and Ole treatments. Table S4. The result of ANOVA test (MS) for exosomes treatments. Table S5. The result of ANOVA test (MS) in exosomes. Table S6. The result of ANOVA test (MS) HFFF2 cell line treated with exosomes. Table S7. Correlation Analysis of MicroRNA Expression in HFFF2‐derived exosomes treated with Fe@C/Ole and Ole compared to exosomes of HFFF2 cells that were treated with exosomes derived from these cells. [file JOCD-24-e70351-s001.docx]

**Supplementary material**

**Table S1.** Gene primer sequences used for qRT-PCR.

| Gene | Gene name | Primer sequence (5′–3′) |
| --- | --- | --- |
| IGF1 | Insulin like growth factor 1 | CAGCAGTCTTCCAACCCAAT  CACGAACTGAAGAGCATCCA |
| IGF1R | Insulin like growth factor1 receptor | ACAACTACGCCCTGGTCATC  CATTCCTTTGGGGGCTTATT |
| COL1A1 | Collagen type I alpha 1 chain | AGCCAGCAGATCGAGAACAT  TCCTTGGCGTTCTTGCTAGT |
| ELN | Elastin | GGTGGCTTAGGAGTGTCTGC  CCAGCAAAAGCTCCACCTAC |
| EGF | Epidermal growth factor | CGCAGGAAATGGGAATTCTA  GCCTTGACCCATTCAGAAAA |
| GAPDH | glyceraldehyde-3-phosphate dehydrogenase | TCCCTGAGCTGAACGGGAAG  GGAGGAGTGGGTGTCGCTGT |

**Table S2.** microRNA primer sequences used for qRT-PCR.

| microRNA | Primer sequence (5′–3′) |
| --- | --- |
| has-miR-29b-3p | ACACTCCAGCTGGGTAGCA  TGGTGTCGTGGAGTCG |
| has-miR-34a-5p | AGGGGGTGGCAGTGTCTTAG  GTGCGTGTCGTGGAGTCG |
| has-let-7d-5p | AGAGGTAGTAGGTTGCAT  GAACATGTCTGCGTATCTC |
| has-let-7e-5p | GGCCTGAGGTAGGAGGTTGT  CAGTGCGTGTCGTGGAGT |
| U6 | CGCTTCGGCAGCACATATAC  TTCACGAATTTGCGTGTCAT |


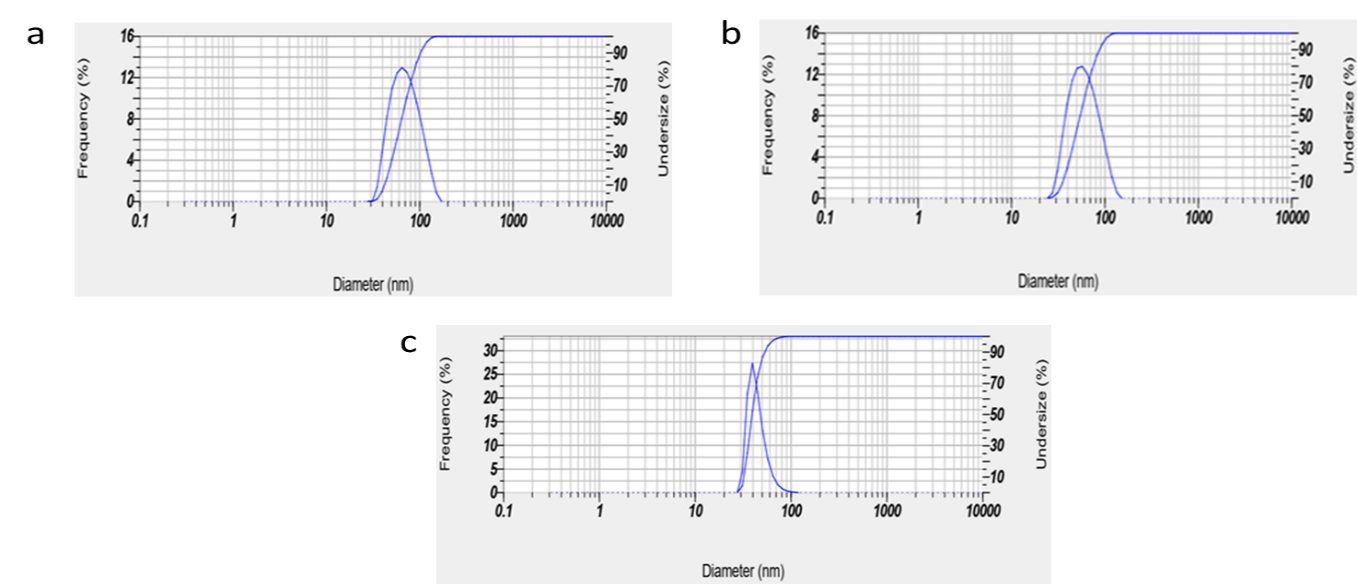


**Figure S1.** DLS to characterize the size of exosomes in hUC-MSCs. **a.** Fe3O4@CQD/Ole, **b.** Ole, **c.** Control group.


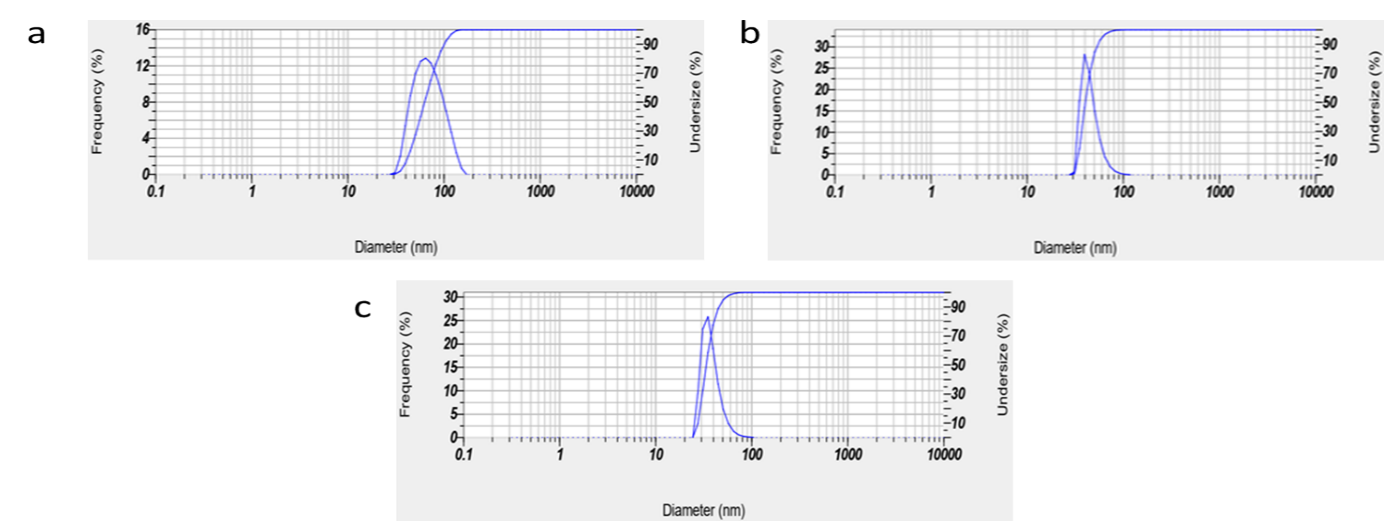


**Figure S2.** DLS to characterize size of exosomes in HFFF2 cell line. **a.** Fe3O4@CQD/Ole, **b.** Ole, **c.** Control group.


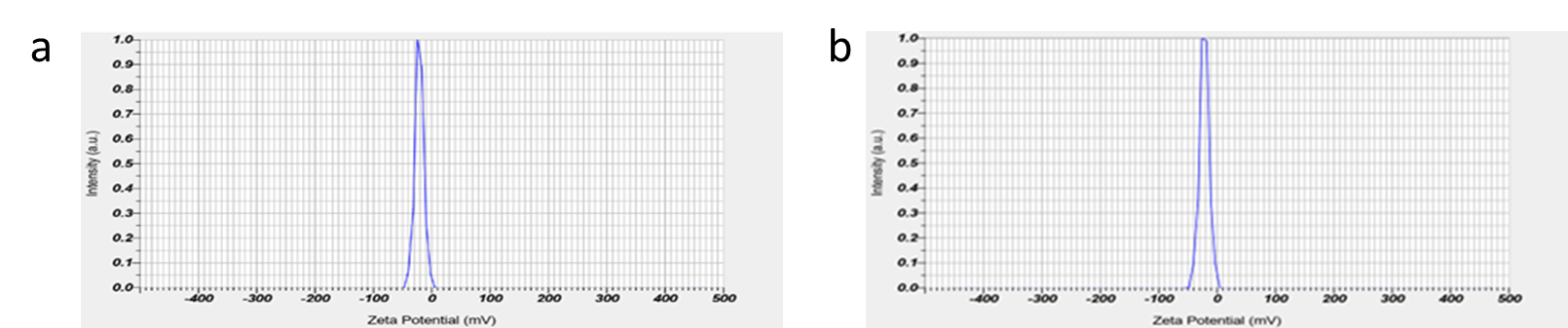


**Figure S3.** Graph of zeta potential under ole treatment in **a.** hUC-MSCs and **b.**) HFFF2 cell line.

**Table S3.** The result of ANOVA test (MS) for Fe@C/Ole and Ole treatments.

|  | Df | IGF1 | | IGF1R | | EGF | | COL1A1 | | ELN | |
| --- | --- | --- | --- | --- | --- | --- | --- | --- | --- | --- | --- |
|  |  | hUC-MSCs | HFFF2 | hUC-MSCs | HFFF2 | hUC-MSCs | HFFF2 | hUC-MSCs | HFFF2 | hUC-MSCs | HFFF2 |
| Treatment | 2 | 3.564^**^ | 4.65^**^ | 5.37^***^ | 4.21^**^ | 6.33ns | 3.77^**^ | 9.34^**^ | 1.16^**^ | 1.87^**^ | 3.65^**^ |
| Error | 6 | 0.54 | 0.43 | 0.82 | 0.47 | 5.98 | 0.45 | 1.65 | 0.14 | 0.13 | 0.13 |
| CV |  | 0.025 | 0.043 | 0.14 | 0.15 | 0.07 | 0.067 | 0.07 | 0.03 | 0.033 | 0.034 |

(ns) nonsignificant, (*) *P ≤ 0.05*, (**) *P ≤ 0.01*, (*** or more) *P ≤ 0.001*

**Table S4.** The result of ANOVA test (MS) for exosomes treatments.

|  | Df | IGF1 | | IGF1R | | EGF | | COL1A1 | | ELN | |
| --- | --- | --- | --- | --- | --- | --- | --- | --- | --- | --- | --- |
|  |  | hUC-MSCs-derived exosomes | HFFF2- derived exosomes | hUC-MSCs-derived exosomes | HFFF2- derived exosomes | hUC-MSCs-derived exosomes | HFFF2- derived exosomes | hUC-MSCs-derived exosomes | HFFF2- derived exosomes | hUC-MSCs-derived exosomes | HFFF2- derived exosomes |
| Treatment | 2 | 5^**^ | 3.76^*^ | 4.66^***^ | 3.91^***^ | 5.11^*^ | 5.77^*^ | 2.89^**^ | 4.22^**^ | 2.89^**^ | 4.22^**^ |
| Error | 6 | 0.48 | 0.65 | 0.21 | 0.13 | 0.66 | 0.74 | 0.23 | 0.44 | 0.23 | 0.44 |
| CV |  | 0.048 | 0.073 | 0.06 | 0.02 | 0.0387 | 0.049 | 0.0387 | 0.0899 | 0.0387 | 0.0899 |

(ns) nonsignificant, (*) *P ≤ 0.05*, (**) *P ≤ 0.01*, (*** or more) *P ≤ 0.001*

**Table S5.** The result of ANOVA test (MS) in exosomes.

|  | Df | hsa-miR-29b-3p | | hsa-miR-34a-5p | | hsa-let-7d-5p | | hsa-let-7e-5p | | |
| --- | --- | --- | --- | --- | --- | --- | --- | --- | --- | --- |
|  |  | hUC-MSCs- exosomes | HFFF2- exosomes | hUC-MSCs- exosomes | HFFF2- exosomes | hUC-MSCs- exosomes | HFFF2- exosomes | hUC-MSCs- exosomes | HFFF2- exosomes |  |
| Treatment | 2 | 1.65^**^ | 1.64^**^ | 1.85^**^ | 5.4^**^ | 1.11^**^ | 1.23^**^ | 2.32^**^ | 2.33^**^ |  |
| Error | 6 | 0.043 | 0.054 | 0.42 | 1.02 | 0.013 | 0.024 | 0.023 | 0.024 |  |
| CV |  | 0.033 | 0.015 | 0.04 | 0.05 | 0.053 | 0.025 | 0.073 | 0.085 |  |

(ns) nonsignificant, (*) *P ≤ 0.05*, (**) *P ≤ 0.01*, (*** or more) *P ≤ 0.001*

**Table S6.** The result of ANOVA test (MS) HFFF2 cell line treated with exosomes.

|  | Df | hsa-miR-29b-3p | | hsa-miR-34a-5p | | hsa-let-7d-5p | | hsa-let-7e-5p | |
| --- | --- | --- | --- | --- | --- | --- | --- | --- | --- |
|  |  | HFFF2treated with hUC-MSCs- exosomes | HFFF2treated with HFFF2- exosomes | HFFF2treated with hUC-MSCs- exosomes | HFFF2treated with HFFF2- exosomes | HFFF2treated with hUC-MSCs- exosomes | HFFF2treated with HFFF2- exosomes | HFFF2treated with hUC-MSCs- exosomes | HFFF2treated with HFFF2- exosomes |
| Treatment | 2 | 1.25^**^ | 1.64^**^ | 2.65** | 3.4* | 1.11** | 1.14** | 2.32** | 2.34** |
| Error | 6 | 0.45 | 0.34 | 0.512 | 0.502 | 0.15 | 0.14 | 0.35 | 0.34 |
| CV |  | 0.03 | 0.05 | 0.05 | 0.05 | 0.02 | 0.015 | 0.032 | 0.055 |

(ns) nonsignificant, (*) *P ≤ 0.05*, (**) *P ≤ 0.01*, (*** or more) *P ≤ 0.001*

**Table S7.** Correlation Analysis of MicroRNA Expression in HFFF2-derived exosomes treated with Fe@C/Ole and Ole compared to exosomes of HFFF2 cells that were treated with exosomes derived from these cells.

| **hsa-miR-29b-3p**  **in exosome content** | **hsa-miR-29b-3p in HFFF2 cells** | | | | |
| --- | --- | --- | --- | --- | --- |
|  |  | Exosome treated with Fe@C/Ole | Exosome treated with Ole | HFFF2-derived  exosome treated with Fe@C/Ole | HFFF2-derived  exosome treated with Ole |
|  | Exosome treated with Fe@C/Ole | - | - | 0.59 | - |
|  | Exosome treated with Ole | - | - | - | 1 |
|  | HFFF2-derived  exosome treated with Fe@C/Ole | 0.59 | - | - | - |
|  | HFFF2-derived  exosome treated with Ole | - | 1 | - | - |
| **hsa- miR-34a-5p**  **in exosome content** | **hsa- miR-34a-5p in HFFF2 cells** | | | | |
|  |  | Exosome treated with Fe@C/Ole | Exosome treated with Ole | HFFF2-derived  exosome treated with Fe@C/Ole | HFFF2-derived  exosome treated with Ole |
|  | Exosome treated with Fe@C/Ole | - | - | 1 | - |
|  | Exosome treated with Ole | - | - | - | 1 |
|  | HFFF2-derived  exosome treated with Fe@C/Ole | 1 | - | - | - |
|  | HFFF2-derived  exosome treated with Ole | - | 1 | - | - |
| **hsa-let-7d-5p**  **in exosome content** | **hsa-let-7d-5p in HFFF2 cells** | | | | |
|  |  | Exosome treated with Fe@C/Ole | Exosome treated with Ole | HFFF2-derived  exosome treated with Fe@C/Ole | HFFF2-derived  exosome treated with Ole |
|  | Exosome treated with Fe@C/Ole | - | - | 0.99 | - |
|  | Exosome treated with Ole | - | - | - | 0.99 |
|  | HFFF2-derived  exosome treated with Fe@C/Ole | 0.99 | - | - | - |
|  | HFFF2-derived  exosome treated with Ole | - | 0.99 | - | - |
| **hsa- let-7e-5p**  **in exosome content** | **hsa- let-7e-5p in HFFF2 cells** | | | | |
|  |  | Exosome treated with Fe@C/Ole | Exosome treated with Ole | HFFF2-derived  exosome treated with Fe@C/Ole | HFFF2-derived  exosome treated with Ole |
|  | Exosome treated with Fe@C/Ole | - | - | 1 | - |
|  | Exosome treated with Ole | - | - | - | 0.97 |
|  | HFFF2-derived  exosome treated with Fe@C/Ole | 1 | - | - | - |
|  | HFFF2-derived  exosome treated with Ole | - | 0.97 | - | - |
